# Agreement and Precision Analyses of Various Estimated Glomerular Filtration Rate Formulae in Cancer Patients

**DOI:** 10.1038/s41598-019-55833-0

**Published:** 2019-12-18

**Authors:** Wiwat Chancharoenthana, Salin Wattanatorn, Somratai Vadcharavivad, Somchai Eiam-Ong, Asada Leelahavanichkul

**Affiliations:** 1 Nephrology Research Unit, Department of Clinical Tropical Medicine, Faculty of Tropical Medicine, Mahidol University, Bangkok, Thailand; 2 Immunology Unit, Department of Microbiology, Faculty of Medicine, Chulalongkorn University, Bangkok, Thailand; 3 Division of Nephrology, Department of Medicine, Faculty of Medicine, Chulalongkorn University, Bangkok, Thailand; 4 Department of Pharmacy Practice, Faculty of Pharmaceutical Sciences, Chulalongkorn University, Bangkok, Thailand; 5 Translational Research in Inflammation and Immunology Research Unit (TRIRU), Department of Microbiology, Faculty of Medicine, Chulalongkorn University, Bangkok, Thailand

**Keywords:** Oncology, Kidney, Chronic kidney disease

## Abstract

The accuracy of the estimated glomerular filtration rate (eGFR) in cancer patients is very important for dose adjustments of anti-malignancy drugs to reduce toxicities and enhance therapeutic outcomes. Therefore, the performance of eGFR equations, including their bias, precision, and accuracy, was explored in patients with varying stages of chronic kidney disease (CKD) who needed anti-cancer drugs. The reference glomerular filtration rate (GFR) was assessed by the ^99m^Tc-diethylene triamine penta-acetic acid (^99m^Tc-DTPA) plasma clearance method in 320 patients and compared with the GFRs estimated by i) the Chronic Kidney Disease Epidemiology Collaboration (CKD-EPI) equation, ii) the unadjusted for body surface area (BSA) CKD-EPI equation, iii) the re-expressed Modification of Diet in Renal Disease (MDRD) study equation with the Thai racial factor, iv) the Thai eGFR equation, developed in CKD patients, v) the 2012 CKD-EPI creatinine-cystatin C, vi) the Cockcroft-Gault formula, and vii) the Janowitz and Williams equations for cancer patients. The mean reference GFR was 60.5 ± 33.4 mL/min/1.73 m^2^. The bias (mean error) values for the estimated GFR from the CKD-EPI equation, BSA-unadjusted CKD-EPI equation, re-expressed MDRD study equation with the Thai racial factor, and Thai eGFR, 2012 CKD-EPI creatinine-cystatin-C, Cockcroft-Gault, and Janowitz and Williams equations were −2.68, 1.06, −7.70, −8.73, 13.37, 1.43, and 2.03 mL/min, respectively, the precision (standard deviation of bias) values were 6.89, 6.07, 14.02, 11.54, 20.85, 10.58, and 8.74 mL/min, respectively, and the accuracy (root-mean square error) values were 7.38, 6.15, 15.97, 14.16, 24.74, 10.66, and 8.96 mL/min, respectively. In conclusion, the estimated GFR from the BSA-unadjusted CKD-EPI equation demonstrated the least bias along with the highest precision and accuracy. Further studies on the outcomes of anti-cancer drug dose adjustments using this equation versus the current standard equation will be valuable.

## Introduction

The coexistence of chronic kidney disease (CKD) and cancer is common due to the increased incidence of cancer in patients with CKD^1^ and the fact that CKD worsens the mortality rate of cancer patients^2^. A precise GFR assessment is fundamental to several aspects of cancer therapy, including chemotherapy dose adjustment, decisions regarding surgery eligibility with perioperative management, and preparation of long-term care. An underestimated GFR in a patient with cancer could lead to inappropriate care, such as in the case of a patient being deemed ineligible for both medical chemotherapy and surgical treatment because their GFR is too low. Conversely, overestimation of the GFR could put a patient at unnecessary risk of drug overdose and unfavorable complications. Because most cancer chemotherapeutic agents are excreted mainly through the kidneys, the accuracy of the estimated glomerular filtration rate (eGFR) in patients with cancer is crucial to balancing treatment efficacy and the risk of adverse events. Although the eGFR calculated from serum creatinine (SCr) is widely used in general practice, overestimation of the GFR due to a patient’s reduced muscle mass and food intake due to malignancy is very common. Despite the increased accuracy of eGFRs obtained by determining the measured GFR (mGFR) using the clearance of exogenous filtration markers, this method has not been widely used due to the necessity of a 24-hour urine collection. Indeed, a standard reference for the eGFR for chemotherapeutic agent dose-adjustments remains undecided. The International Society of Geriatric Oncology preferred eGFRs using the Modification of Diet in Renal Disease (MDRD) equation over the Cockcroft-Gault equation for patients over 65 years old. Nevertheless, the equation from the Chronic Kidney Disease Epidemiology Collaboration (CKD-EPI) in a recent large-scale, retrospective study appeared to be superior to the MDRD equation for the eGFR assessment in patients with cancer^3^.

In general, a consensus of the international guideline group of KDIGO (Kidney Disease: Improving Global Outcomes) recommends creatinine-based equations for initial testing, with other confirmatory tests for the estimation of kidney function in CKD patients^4^. Similarly, the Society of Geriatric Oncology guidelines prefer mGFR in anti-cancers that are mainly excreted through the kidney (or with apparent nephrotoxicity) or in cases of possibly inaccurate eGFRs^5^. It is important to recognize that both the MDRD and the CKD-EPI equations were developed using data from CKD patients, from which patients with malignancy are excluded^6–11^. Patients with malignancy, in contrast to those with CKD alone, mostly suffer from more severe sarcopenia, resulting in lower SCr (due to less creatinine production) and overestimated GFRs. As such, accurately estimated renal function is likely necessary for the proper adjustment of cancer chemotherapy. Although the MDRD and CKD-EPI equations are widely used to predict the GFR in the United States and Europe, some corrections are necessary for other ethnic groups, as is the case for the coefficient factor for the isotope-dilution mass-spectrometry (IDMS) tracer in the re-expressed MDRD equation proposed as 1.129 for the Thai population^10^. However, the validation of the eGFR derived from these equations in Thai patients with cancer has not yet been investigated.

Moreover, serum cystatin C (CysC) is considered a potential replacement for SCr as a filtration marker, but the correlation between the eGFRs derived from CysC and SCr are still uncertain^12–14^. In addition, increased serum CysC levels found in both solid and hematologic malignancies are likely related to the tumor’s nature as a cysteine protease inhibitor^15–17^. No studies have examined whether an equation based on serum CysC would improve GFR estimation in cancer patients compared to the estimates obtained by other equations. Therefore, the aim of the present study was to investigate the agreement and precision of the currently published eGFR formulae, including the CKD-EPI^7^, the body surface area (BSA)-unadjusted CKD-EPI, the re-expressed MDRD study equation with the Thai racial factor^10,18^, the Thai eGFR^10^, the 2012 CKD-EPI creatinine-cystatin C^13^, the Cockcroft-Gault^19^, and the most recent cancer patient-derived eGFR equation by Janowitz and Williams^3^, compared to the standard GFR measurement by ^99m^Tc-DTPA.

## Materials and Methods

### Study design and patient selection

The study was performed in compliance with the Helsinki Declaration. All participants were informed and provided written informed consent to participate in this study, which was approved by the Human Research Ethics Committee of Chulabhorn Research Institute (No. 017/2559) and local institutional review boards. The inclusion criteria were adults aged 18–70 years old with the following conditions: (i) pathologically or cytologically proven solid or hematologic malignancy with a performance status according to the Eastern Cooperative Oncology Group (ECOG) of 0–1 and ii) CKD in stable condition at various stages (G1–G5) according to the KDIGO criteria^4^. The exclusion criteria were as follows: i) history of active medical or surgical treatment for related malignancy within the past 6 months; (ii) acute deterioration of malignancy or related complications, including gastrointestinal bleeding, infection, severe malnutrition with an edematous state, acute kidney injury superimposed on CKD, congestive heart failure, and arterial or venous thrombosis; (iii) dialysis dependence; (iv) amputation; (v) breastfeeding or pregnancy; (vi) end-of-life status; (vii) current hospitalization; and (viii) current use of medications with SCr interference, including ascorbic acid, corticosteroids, trimethoprim, cimetidine, flucytosine, methyldopa, and levodopa.

### Reference GFR measurement

The reference GFR in the present study was determined by the ^99m^Tc-diethylene triamine penta-acetic Acid (^99m^Tc-DTPA) plasma clearance method with a radiopurity of >95% and the percentage bound to plasma protein <5%. The reference GFR by ^99m^Tc-DTPA plasma clearance was read by a radiologist who was blinded to the clinical data. All participants were measured for plasma radioactivity of ^99m^Tc-DTPA at 5, 30, 60, 120, 180, and 240 minutes after a single intravenous bolus of ^99m^Tc-DTPA, following the institutional protocol. Then, plasma radioactive activities were plotted as a function of time to create a time–activity curve to calculate the GFR normalized by BSA^20^ as well as the measured GFR (mGFR) according to the following equation (D, dosage of drug injected; *t*, time of blood sampling):^21^

$${\rm{GFR}}=\frac{{\rm{D}}}{{\rm{area}}\,{\rm{under}}\,{\rm{time}}-{\rm{activity}}\,{\rm{curve}}}=\frac{{\rm{D}}}{{\int }_{0}^{\infty }c(t){\rm{d}}t}$$

### Measurements of serum creatinine, serum cystatin C, and the 24-hour urine creatinine clearance

The serum creatinine (SCr) of individuals was evaluated by an enzymatic assay with the COBAS INTRGRA^®^ 400 plus autoanalyzer (Roche Diagnostic, Indianapolis, IN, USA) adjusted with a traceable high-level IDMS reference. Serum cystatin C (CysC) was measured by an automated particle-enhanced turbidimetric immunoassay (PETIA) on an ARCHITECT AEROSET analyzer (Abbott Diagnostics, IL, USA). The coefficient of variation for the serum CysC assay was 2.1%. Both SCr and serum CysC were measured within 30 days of the ^99m^Tc-DTPA–reference GFR measurement. No patient-identifiable data were used. Anonymized data included age, sex, height, weight, BSA, blood pressure, SCr, serum CysC, and serum albumin, all measured on the same day. Body composition was assessed by bioimpedance analysis using a Body Composition Analyzer (InBody 230, Biospace Corp., Seoul, Korea).

### Evaluation of renal function by the eGFR

Seven different commonly used methods of GFR estimation were tested in this study, including the re-expressed MDRD study equation with the Thai racial factor, the CKD-EPI equation with and without the BSA adjustment, and the 2012 CKD-EPI creatinine-cystatin C, Cockcroft-Gault, Thai eGFR, and Janowitz and Williams equations, as shown in Table 1. It is interesting to note that the estimated GFR calculated from all of the selected equations, except the Janowitz and Williams equations and Cockcroft-Gault equation, are already adjusted for BSA by intrinsic design; therefore the unit is already expressed as “mL/min/1.73 m^2^” without the necessity for BSA adjustment in calculated eGFR values”. The units of the Janowitz and Williams equations and estimated creatinine clearance by Cockcroft-Gault equation express as “mL/min”^3,19^. In addition, a BSA-unadjusted GFR for those equations are calculated by the following formulae: BSA-unadjusted GFR (mL/min) = eGFR (mL/min/1.73 m^2^) x [BSA (m^2^)/1.73].

**Table 1:** Estimated glomerular filtration rate (eGFR) equations used in the present study.

eGFR equations [ref.]	Gender	SCr	Formulas
CKD-EPI^7^	Female	Cr_Enz_ ≤0.7 mg/dL	144 × (Cr_Enz_/0.7)^−0.329^ × (0.993)Age
	Female	Cr_Enz_ >0.7 mg/dL	144 × (Cr_Enz_/0.7)^−1.209^ × (0.993)Age
	Male	Cr_Enz_ ≤0.9 mg/dL	141 × (Cr_Enz_/0.9)^−0.411^ × (0.993)Age
	Male	Cr_Enz_ >0.9 mg/dL	141 × (Cr_Enz_/0.9)^−1.209^ × (0.993)Age
BSA-unadjusted CKD-EPI	—	Cr_Enz_	eGFR (from CKD-EPI, in mL/min/1.73 m^2^) × BSA (in m^2^) /1.73
Re-expressed MDRD study with the Thai racial factor^10^	—	Cr_Enz_	175 × (Cr_Enz_)^−1.154^ × (Age)^−0.203^ × (0.742 if female) × (1.129 if Thai)
Thai eGFR^10^	—	Cr_Enz_	375.5 × (Cr_Enz_)^−0.848^ × (Age)^−0.364^ × (0.712 if female)
2012 CKD-EPI creatinine-cystatin C^13^	—	—	135 × min(Cr_Enz_/*κ*, 1)^*α*^ × max(Cr_Enz_/*κ*, 1)^−0.601^ × min(CysC/0.8, 1)^−0.375^ × max(CysC/0.8, 1)^−0.711^ × 0.995^Age^ [×0.969 if female] [×1.08 if black] where *κ* is 0.7 for females and 0.9 for males, *α* is −0.248 for females and −0.207 for males, min indicates the minimum of Scr/*κ* or 1, and max indicates the maximum of Scr/*κ* or 1.
Cockcroft-Gault^19^	—	Cr_Enz_	[(140–Age) × BW/Cr_Enz_ × 72] × (0.85 if female)
Janowitz & Williams^3^	—	—	$$\begin{array}{c}\sqrt{{\rm{GFR}}}=1.8140+0.01914{\rm{Age}}+4.7328{\rm{BSA}}-3.7162\,\log ({{\rm{Cr}}}_{{\rm{Enz}}})-0.9142\,\log \,{({{\rm{Cr}}}_{{\rm{Enz}}})}^{2}\\ \,+1.0628\,\log \,{({{\rm{Cr}}}_{{\rm{Enz}}})}^{3}-0.0297{\rm{Age}}\times {\rm{BSA}}+(0.0202+0.0125{\rm{Age}})[{\rm{if}}\,{\rm{Sex}}={\rm{male}}]\end{array}$$

Age units are years.

BSA, body surface area (with units of m^2^, calculated using the DuBois equation); BW, body weight (with units of kilograms); CKD-EPI, Chronic Kidney Disease Epidemiology Collaboration; Cr_Enz_, serum creatinine measured by enzymatic method (with units of mg/dL); CysC, serum cystatin C (with units of mg/L); eGFR, estimated glomerular filtration rate; MDRD, Modification of Diet in Renal Disease; ref., reference; SCr, serum creatinine.

### Statistical analysis

The baseline characteristics of the patients are presented as the mean ± standard deviation (SD). Other data are presented as median ± interquartile ranges (IQR). Student’s *t*-test or the Mann–Whitney *U* test and the χ^2^ or Fischer’s exact test were conducted to compare continuous variables and categorical variables, respectively. Bland-Altman plots were used to assess the agreement between the reference GFR and eGFR^22^. The difference between the reference GFR and eGFR (reference GFR minus eGFR) was also calculated. The performances of the eGFR equations were evaluated for bias and precision. Bias measurements were expressed as the mean error (ME)^23^. Meanwhile, precision was defined as the standard deviation (SD) of the mean absolute difference^24^. Accuracy was defined as the root-mean square error (RMSE), which was calculated according to the following formula (n represents the sample size):^25^

$${\rm{RMSE}}=\sqrt{\frac{{\sum }_{i=1}^{{\rm{n}}}{({{\rm{P}}}_{i}-{{\rm{O}}}_{i})}^{2}}{{\rm{n}}}}$$

In addition to the RMSE, the accuracy of the equations was also calculated using the percentage of the eGFR falling within the range of 10%, 15%, and 30% of the reference GFR. Statistical analyses were performed using STATA version 13.1 (StataCorp., College Station, TX, USA). A *p-*value < 0.05 was considered a statistically significant difference.

## Results

### Participants’ baseline characteristics

The patient characteristics are summarized in Table 2. A total of 320 cancer patients were studied, of which 299 (93.4%) and 21 (6.6%) patients had solid malignancy and hematologic malignancy, respectively. The median ^99m^Tc-DTPA clearance (the reference GFR) was 50.4 mL/min/1.73 m^2^ (interquartile range [IQR] from 32.6 to 86.6 mL/min/1.73 m^2^), with almost 80% of patients categorized with stages G1–G3b of chronic kidney disease (CKD) according to the KDIGO classification. Notably, there was no participant with an extreme GFR (i.e., greater than 150 mL/min/1.73 m^2^) during the observation period. The average body mass index (BMI) and body surface area (BSA) were 21.6 ± 3.1 kg/m^2^ and 1.68 ± 0.2 m^2^, respectively. The mean serum creatinine (SCr) was 2.5 ± 1.6 mg/dL (95% confidence interval [CI] of 1.48 to 3.29 mg/dL). In addition, the median muscle mass and soft lean mass were 22.5 ± 5.8 and 39.6 ± 8.7 kg, respectively, which was significantly lower than those in normal-weight and lean populations^26^ (*p* < 0.01).

**Table 2:** Baseline characteristics of participants.

Characteristics	All (n = 320)	Female (n = 154)	Male (n = 166)
Age (years)	55 ± 16.4	52 ± 15.3	57 ± 13.8
Weight (kg)	50.5 ± 13.8	48 ± 12.1	53 ± 14.5
Height (m)	1.65 ± 0.2	1.57 ± 0.1	1.68 ± 0.2
BMI (kg/m^2^)	21.6 ± 3.1	18.8 ± 1.3	20.3 ± 3.6
BSA (m^2^)	1.68 ± 0.2	1.63 ± 0.2	1.72 ± 0.2
Muscle mass (kg)	22.5 ± 5.8	19.8 ± 3.8	21.9 ± 10.8
Soft lean mass (kg)	39.6 ± 8.7	38.6 ± 4.4	41.3 ± 8.0
Body fat mass (kg)	10.4 ± 9.6	10.7 ± 8.1	11.8 ± 4.2
Fat free mass (kg)	41.3 ± 7.3	40.8 ± 2.7	41.4 ± 7.7
Proteinuria (g/day)	0.42 ± 0.5	0.42 ± 0.3	0.43 ± 0.6
Blood urea nitrogen (mg/dL)	27.3 ± 19.4	25.7 ± 18.2	30.8 ± 20.4
Serum creatinine (mg/dL)	2.5 ± 1.6	2.4 ± 1.2	2.6 ± 1.7
Serum albumin (g/dL)	2.6 ± 1.7	2.5 ± 1.8	2.6 ± 1.5
Mean arterial blood pressure (mmHg)	72.6 ± 11.6	71.8 ± 12.4	72.1 ± 14.2
Hypertension (n, (%))	33 (10.3)	14 (9.1)	19 (11.4)
Reference GFR (mL/min/1.73 m^2^)	60.5 ± 33.4	54.6 ± 31.8	62.3 ± 28.7
Reference GFR by category of CKD (n, (%))			
G1 (eGFR ≥ 90 mL/min/1.73 m^2^)	77 (24.1)	35 (22.7)	42 (25.3)
G2 (eGFR 60–89 mL/min/1.73 m^2^)	62 (19.4)	34 (22.1)	28 (16.9)
G3a (eGFR 45–59 mL/min/1.73 m^2^)	49 (15.3)	22 (14.3)	27 (16.3)
G3b (eGFR 30–44 mL/min/1.73 m^2^)	69 (21.5)	31 (20.1)	38 (22.9)
G4 (eGFR 15–29 mL/min/1.73 m^2^)	38 (11.9)	20 (13.0)	18 (10.8)
G5 (eGFR < 15 mL/min/1.73 m^2^)	25 (7.8)	12 (7.8)	13 (7.8)
Types of primary malignancy (n, (%))			
Solid malignancy	299 (93.4)	146 (94.8)	153 (92.2)
Hematologic malignancy	21 (6.6)	8 (5.2)	13 (7.8)
Stages of malignancy (n, (%))			
Stage 1	164 (51.3)	87 (56.5)	77 (46.4)
Stage 2	139 (43.4)	61 (39.6)	78 (47.0)
Stage 3	17 (5.3)	6 (3.9)	11 (6.6)
Stage 4	0 (0)	0 (0)	0 (0)

Data are shown as the mean ± SD unless otherwise specified.

BMI, body mass index; BSA, body surface area; CKD, chronic kidney disease.

### Difference between the reference GFR and the GFR estimated from various equations

The performances of seven published models of estimated GFR (eGFR) (see Methods) in patients with cancer were compared with those of the reference GFR. Among them, the eGFR from the BSA-unadjusted CKD-EPI equation demonstrated the greatest accuracy according to the root-mean square error (RMSE) (6.15 mL/min; 95% CI, 5.82 to 7.61 mL/min) with the least bias (mean error [ME], 1.06 mL/min; 95% limits of agreement, −10.83 to 12.95 mL/min). The novel model of the Janowitz and Williams equation was the third most accurate and least biased model for the eGFR; the RMSE and ME were 8.96 mL/min (95% CI, 6.96 to 9.77 mL/min) and 2.03 mL/min (95% limits of agreement, −15.11 to 19.16 mL/min), respectively. For the re-expressed MDRD study equation with the Thai racial factor, the RMSE and ME were 15.97 mL/min (95% CI, 14.96 to 17.38 mL/min) and −7.70 mL/min (95% limits of agreement, −35.17 to 19.78 mL/min), respectively. For the Thai eGFR equation, the RMSE and ME were 14.16 mL/min (95% CI, 13.08 to 16.31 mL/min) and −8.73 mL/min (95% limits of agreement, −31.36 to 13.89 mL/min), respectively. Notably, the 2012 CKD-EPI creatinine-cystatin C equation demonstrated the most bias with the least accuracy compared with the other equations **(**Table 3). We also determined the effect of adjusting BSA on various estimated GFR accuracy (RMSE) as shown in Table 4.

**Table 3:** The means of the reference GFR and the eGFRs calculated by the different eGFR equations. The bias between the mean eGFR and the reference GFR and the range of the bias are shown.

Estimated GFR models	GFR* (n = 320)	Bias		Precision	Accuracy			
		ME	95% limits of agreement	SD of bias	RMSE	P_10_ (%)	P_15_ (%)	P_30_ (%)
Reference GFR	50.4 (32.6–86.5), 7.9–142.3	—	—	—	—	—	—	—
CKD-EPI	55.7 (35.8–84.6), 9.3–130.2	−2.68	−16.18 to 10.83	6.89	7.38	51.88	72.81	96.25
BSA-unadjusted CKD-EPI	51.4 (33.1–81.6), 7.7–143.8	1.06	−10.83 to 12.95	6.07	6.15	71.88	87.50	99.06
Re-expressed MDRD study with the Thai racial factor	57.3 (37.9–85.8), 10.0–196.4	−7.70	−35.17 to 19.78	14.02	15.97	37.19	54.69	86.25
Thai eGFR	62.9 (43.5–83.5), 17.0–159.3	−8.73	−31.36 to 13.89	11.54	14.16	25.94	35.31	58.13
2012 CKD-EPI creatinine-cystatin C	36.3 (24.2–57.6), 3.3–192.1	13.37	−27.49 to 54.23	20.85	24.74	17.19	26.56	55.31
Cockcroft-Gault	51.1 (33.2–74.4), 8.3–158.4	1.43	−19.30 to 22.17	10.58	10.66	44.69	65.63	91.25
Janowitz & Williams	55.0 (35.3–76.7), 5.7–128.4	2.03	−15.11 to 19.16	8.74	8.96	54.69	77.50	95.00

CKD-EPI, Chronic Kidney Disease Epidemiology Collaboration; eGFR, estimated glomerular filtration rate; MDRD, Modification of Diet in Renal Disease; ME, mean error (negative values signify overestimation); P_n_, percentage of participants with an eGFR within ± n % of the reference GFR; RMSE, root-mean square error; SD, standard deviation.

*Data presented as median (IQR), range with the units of mL/min/1.73 m^2^ (except the BSA-unadjusted CKD-EPI, Cockcroft-Gault, and Janowitz & Williams which demonstrated as the units of mL/min).

**Table 4:** Comparisons between the accuracy (determined by the root-mean square error (RMSE)) of various estimated glomerular filtration rate (eGFR) model (BSA-adjusted vs. BSA-unadjusted) equations and the reference GFR.

Methods of GFR assessment	Root mean square error (mL/min)	
	BSA-adjusted	BSA-unadjusted
Reference	—	—
CKD-EPI	7.38	6.15
Re-expressed MDRD study with the Thai racial factor	15.97	22.07
Thai eGFR	14.16	19.75
2012 CKD-EPI creatinine-cystatin C	24.74	23.53
Cockcroft-Gault	11.14	10.66
Janowitz & Williams	8.96	11.82

BSA, body surface area; CKD-EPI, Chronic Kidney Disease Epidemiology Collaboration; eGFR, estimated glomerular filtration rate; MDRD, Modification of Diet in Renal Disease.

### Diagnostic performance of various estimated GFR equations compared to the reference GFR

**Figure 1:**
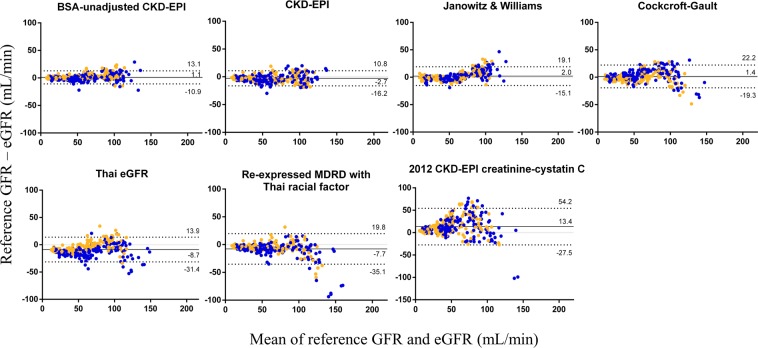
Bland-Altman plots of estimated GFR (eGFR) versus the reference GFR for each model’s equation are shown. The mean of the reference GFR and eGFR was plotted against the difference between the two. Positive and negative differences indicate under- and overestimation, respectively. The plots are shown in ascending order of the precision of the eGFR from top left to bottom right, where the precision is calculated by the root-mean-squared error. The solid black line on each plot represents the mean of the difference, the solid gray line marks the line of identity, and the dashed line is drawn at the mean ± 1.96 times the standard deviation of the difference. Points are colored by sex (blue and orange represent female and male, respectively). BSA, body surface area; CKD-EPI, Chronic Kidney Disease Epidemiology Collaboration; MDRD, Modification of Diet in Renal Disease.

The agreement between the measurements by Bland-Altman and residual plots indicated that the BSA-unadjusted CKD-EPI equation showed the most accurate, least biased, and least heteroscedastic results, i.e., the most constant variance in different subpopulations, compared to those from the other equations **(**Fig. 1**)**. Regarding sex differences, the BSA-unadjusted CKD-EPI equation demonstrated the most homogeneity between male and female patients compared to the other eGFR equations. Notably, the Thai eGFR equation clearly demonstrated an overestimation of GFR in males in comparison to that in females **(**Fig. 1**)**.

**Figure 2:**
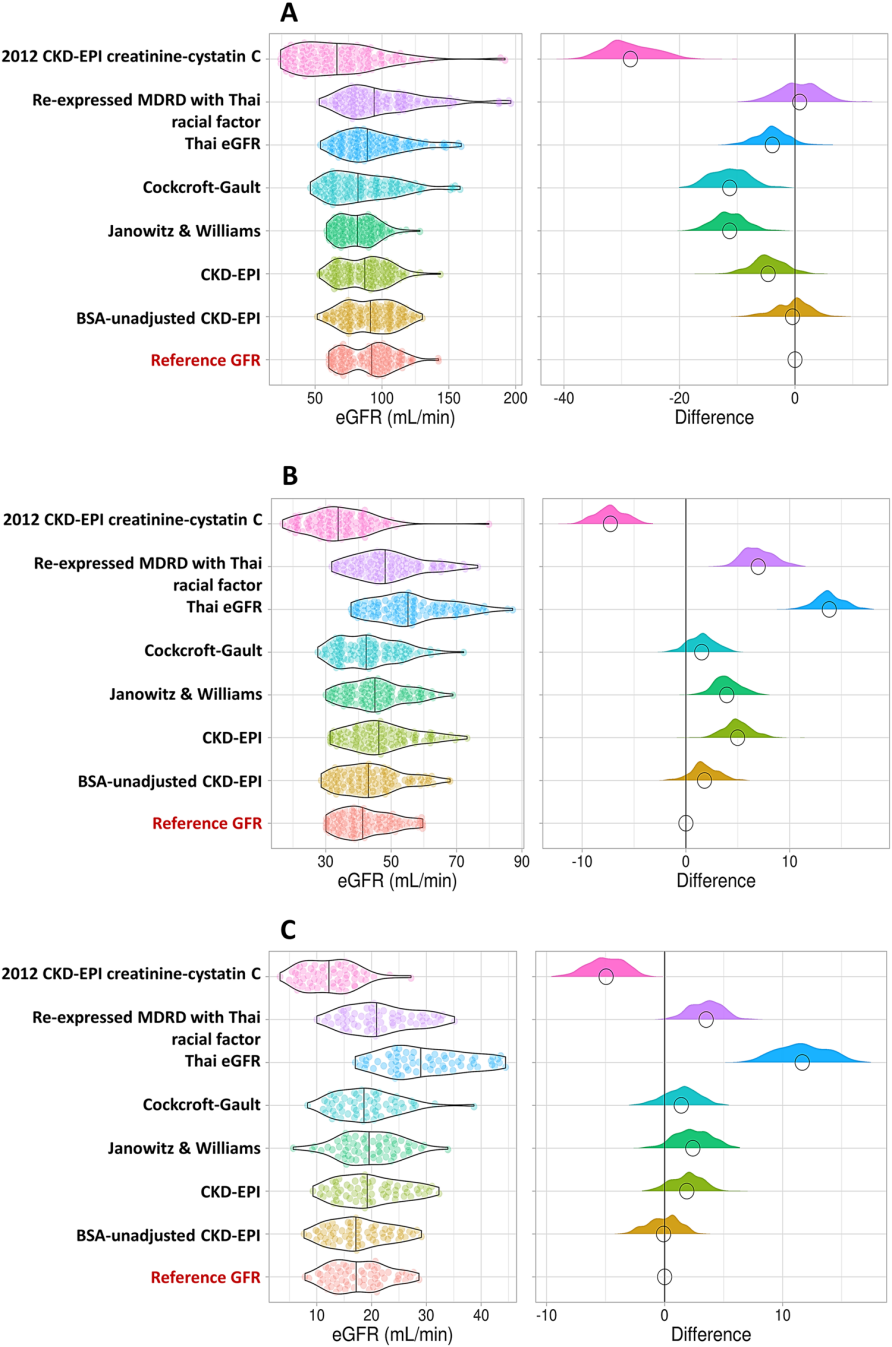
Violin plot of the differences between the model equations’ outcomes and the reference GFR according to the GFR ranges: **(A)** GFR ≥60 mL/min, **(B)** GFR 30–59 mL/min, and **(C)** GFR <30 mL/min. The solid black lines in the left panels refer to the medians of the eGFR for each eGFR model, while the black circles on the right panels represent the medians of the difference for each eGFR model. Positive and negative differences indicate over- and underestimation, respectively. BSA, body surface area; CKD-EPI, Chronic Kidney Disease Epidemiology Collaboration; MDRD, Modification of Diet in Renal Disease.

We also investigated the utility of these eGFR equations with the reference GFR in the CKD population grouped according to GFR range as following: i) GFR ≥60 mL/min, ii) GFR 30–59 mL/min, and iii) GFR <30 mL/min. As shown in Fig. 2, the BSA-unadjusted CKD-EPI equation was the least biased model for estimating the GFR, illustrated by the violin plot in all CKD categories. Although the Cockcroft-Gault equation was the second least biased model calculated by ME **(**Table 2**)**, it underestimated the GFR, particularly in patients with GFR ≥60 mL/min **(**Fig. 2A**)**. Similar to the BSA-unadjusted CKD-EPI equation, both the Janowitz and Williams equation and the CKD-EPI equation yielded estimates that were compatible with the reference GFR (i.e., less different from the reference GFR) in all GFR ranges. Meanwhile, both the Thai eGFR equation and the re-expressed MDRD study equation with the Thai racial factor demonstrated overestimation of the GFR in advanced CKD **(**Fig. 2B,C**)**.

### Sensitivity and specificity of the eGFR equations for identifying various CKD stages

The performances of the published models were also analyzed according to the KDIGO classification (CKD stage G1–G5), as shown in Table 5. The re-expressed MDRD study equation with the Thai racial factor demonstrated the highest sensitivity (91.7%) and specificity (100%) in CKD stage G1. Meanwhile, the BSA-unadjusted CKD-EPI equation was the model with the best performance across CKD stages G2–G5. Interestingly, most of the published models showed less sensitivity and specificity in advanced CKD. It should be noted that only the CKD-EPI equation, regardless normalization by BSA, was suitable for determining CKD stage G5 based on the eGFR. In fact, the greatest sensitivity (89.7%) and specificity (100%) for CKD stage G5 were demonstrated by the BSA-unadjusted CKD-EPI equation.

**Table 5:** The performances of published estimated glomerular filtration rate (GFR) models for chronic kidney disease determination.

Estimated GFR models	Chronic kidney disease stage				
	G1	G2	G3	G4	G5
CKD-EPI					
Sensitivity	85.5	79.0	84.8	87.4	88.6
Specificity	100.0	75.0	44.4	100.0	100.0
PPV	100.0	89.0	95.2	100.0	100.0
NPV	24.6	13.0	18.2	11.1	25.0
BSA-unadjusted CKD-EPI					
Sensitivity	80.6	90.7	89.9	97.3	89.7
Specificity	100.0	62.5	55.6	100.0	100.0
PPV	100.0	94.2	96.1	100.0	100.0
NPV	26.3	50.0	31.3	50.0	40.0
Re-expressed MDRD study with the Thai racial factor					
Sensitivity	91.7	90.7	87.2	83.8	52.1
Specificity	100.0	62.5	55.6	100.0	100.0
PPV	100.0	94.2	95.9	100.0	100.0
NPV	45.5	50.0	26.3	14.3	15.4
Thai eGFR					
Sensitivity	76.4	88.9	70.6	32.4	4.3
Specificity	100.0	62.5	66.7	100.0	100.0
PPV	100.0	94.1	96.3	100.0	100.0
NPV	22.7	45.5	15.8	3.8	8.3
2012 CKD-EPI creatinine-cystatin C					
Sensitivity	28.6	27.4	65.3	72.2	68.0
Specificity	100.0	37.5	22.2	100.0	100.0
PPV	100.0	73.3	91.7	100.0	100.0
NPV	18.3	62.5	46.5	22.5	24.2
Cockcroft-Gault					
Sensitivity	72.2	63.0	92.7	91.9	69.6
Specificity	100.0	37.5	44.4	100.0	100.0
PPV	100.0	87.2	95.3	100.0	100.0
NPV	20.0	13.0	33.3	25.0	22.2
Janowitz & Williams					
Sensitivity	55.6	98.2	92.7	91.9	30.4
Specificity	100.0	37.5	44.4	100.0	100.0
PPV	100.0	91.3	95.3	100.0	100.0
NPV	13.5	75.0	33.3	25.0	11.1

Data are represented as percentages (%).

CKD-EPI, Chronic Kidney Disease Epidemiology Collaboration; MDRD, Modification of Diet in Renal Disease; PPV, positive predictive value; NPV, negative predictive value.

## Discussion

Our study showed that the body surface area (BSA)-unadjusted CKD-EPI equation showed the best performance for GFR estimation in terms of both precision and accuracy, followed (in order) by the CKD-EPI equation as well as the Janowitz and Williams equation for patients with cancer, the Cockcroft-Gault equation, and the Thai eGFR equations. Meanwhile, the 2012 CKD-EPI creatinine-cystatin C equation was the least precise and the least accurate eGFR equation in cancer patients as determined by the standard deviation of the absolute difference and root-mean square error (RMSE), respectively.

GFR is currently the standard measurement for determining renal function^27^, and patients with cancer commonly present with impaired renal function^28^. At present, there are three most commonly used formulae in oncology worldwide—the Cockcroft-Gault, the MDRD study, and the CKD-EPI equations^7,18,19^—as well as the Thai eGFR equation, which is being adopted in practice nationwide^10^. While the CKD-EPI equation is recommended for use in routine clinical practice by the KDOQI and the National Kidney Foundation (NKF), most cancer centers use the MDRD study equation, following the International Society of Geriatric Oncology recommendation^5^. Nevertheless, the CKD-EPI equation is more accurate than the MDRD study equation in patients with reduced muscle mass, as eGFRs of 45–60 mL/min/1.73 m^2^ estimated by the MDRD study equation might be estimated as above 60 mL/min/1.73 m^2^ by the CKD-EPI equation^11^. Moreover, Asians have been shown to have a higher percentage of body fat for the same level of BMI than Caucasians, suggesting lower levels of muscle mass;^29^ this suggests ethnic interference and the necessity for robust validation of eGFRs in patients with cancer.

In our study, the BSA-unadjusted CKD-EPI equation was the least biased equation **(**Figs. 1 and 2**)**; it was less biased than the Cockcroft-Gault equation and the re-expressed MDRD study equation with the Thai racial factor. Although the Cockcroft-Gault equation demonstrated the second least bias of the eGFR equations (mean error 1.43 mL/min), the precision and accuracy were less than the those of BSA-unadjusted CKD-EPI, CKD-EPI, and Janowitz and Williams equations **(**Table 3**)**. The re-expressed MDRD study equation with the Thai racial factor, a preferable equation for CKD in the Thai population^10^, showed widest bias in eGFRs < 60 mL/min with a tendency of overestimation **(**Fig. 2**)**, possibly due to sarcopenia in patients with cancer^30^. Indeed, the participants in the present study had an 8.5% lower mean muscle mass compared to those of patients with HIV infection (22.5 ± 5.8 vs. 24.6 ± 5.6 kg, *p* < 0.001), another chronic illness population^31^. Additionally, the BSA-unadjusted CKD-EPI equation would be more applicable than the re-expressed MDRD study equation for calculation of eGFR in cancer patients with higher sensitivity and specificity in CKD determination, particularly in patients with CKD stage G2–G5 **(**Table 5**)**. However, the use of BSA in corporation with eGFR formulas should be interpreted with caution particularly in CKD stages of KDIGO because the unit of eGFR in KDIGO naturally presents as mL/min/m^2^ ^4^. In other words, there must be no difference between BSA-adjusted equations and BSA-unadjusted equations in term of the KDIGO guideline.

Although the 2012 CKD-EPI creatinine-cystatin C equation was favorable in conditions of low SCr production, such as in the case of loss of muscle mass from limb amputations or neurological diseases^32^, the 2012 CKD-EPI creatinine-cystatin C eGFRs had low precision and accuracy in our results, possibly due to the lack of patients with cancer during the standardization of this equation^13,33^. Interestingly, the Thai eGFR equation demonstrated better performance than the 2012 CKD-EPI creatinine-cystatin C equation and the re-expressed MDRD study equation with the Thai racial factor, possibly due to the increased generalizability to the CKD population of the Thai eGFR equation and/or the different methods used for the reference GFR determination^10^. A further validation study in patients with cancer might be necessary to identify proper serum CysC-based and/or SCr-based eGFR equations for the Thai population. Moreover, the spread of bias among the BSA-unadjusted CKD-EPI, CKD-EPI, and Janowitz and Williams equations from the reference GFR was evenly distributed **(**Fig. 1**)** despite the increased deviation from the reference in patients with eGFR <60 mL/min with the Janowitz and Williams equation **(**Fig. 2B,C**)**. This phenomenon might be explained by the low sensitivity for advanced CKD stage with the Janowitz and Williams equation. Although the Janowitz and Williams equation is a somewhat sophisticated mathematical formula and is weak in its assessment of advanced CKD, it was impressive in assessments of early-stage CKD and is available as an online calculation tool (http://tavarelab.cruk.cam.ac.uk/JanowitzWilliamsGFR/)^34^.

Given the validation of several common eGFR calculations, the CKD-EPI equation (regardless of BSA adjustment) is the most appropriate for determining the CKD stage in patients with malignancy (malnourishment or severe emaciation are common). Our findings support the 2016 cancer chemotherapy guidelines for treatment of renal injury, which states that i) eGFR (or creatinine clearance) without correcting BSA is used for drugs that the doses are fixed (BSA independent) and ii) eGFR (or creatinine clearance) corrected for BSA is used for drugs that the dose depends on BSA^35^. Although there is currently no guideline consensus which method of eGFR is preferred in cancer patients, our findings are consistent with the most recent study by Janowitz and colleagues^3^, which demonstrate better predictive performance of the BSA-unadjusted CKD-EPI over the CKD-EPI equation. While the CKD-EPI equation is recommended for use in routine clinical practice by the KDOQI and the National Kidney Foundation (NKF), the CKD-EPI equation showed less accuracy compared with the BSA-unadjusted CKD-EPI in the present study. This paradox could be explained by the fact that the CKD-EPI equations included populations with mean BSA of 1.93 ± 0.2 m^2^ and BMI of 28 ± 6 kg/m^2^, reflecting the large number of overweight participants in the CKD-EPI study^7^. Interestingly, Levey *et al*.^7^ also reported the mean measured GFR of their studied CKD patients of 68 mL/min/1.73 m^2^ and the mean BSA-unadjusted measured GFR of 75.9 mL/min. Accordingly, the difference of 7.9 mL/min was found after reversing the BSA indexing process among their studied population. In the present study, the CKD-EPI equation showed greater performance over the Janowitz and Williams equation, particularly in CKD with GFR < 30 mL/min, possibly due to (i) the ethnic difference^10^, (ii) the higher proportion of patients with low muscle mass (and BMI) in our study, iii) the difference in reference eGFR (^99m^Tc-DTPA plasma clearance in the present study versus three different time points of chromium-51 EDTA (^51^Cr-EDTA) administration in the other study) and iv) the inclusion criteria including both solid and hematologic malignancy in the present study^3^.

There were several limitations in our study. First, the gold standard renal inulin clearance was not included in the present study. Although the ^99m^Tc-DTPA method may overestimate GFR, particularly in patients with lower BMI^36^, the comparable inulin method for CKD patients has been mentioned in a large study^37^. Second, the performance status of most participants was good (ECOG 0–1) due to ethical restrictions. Patients with cachexia might have displayed more deviations in GFR. Third, a small number of patients with paraproteinemia—a disease with low SCr—were included in the present study. However, the exclusion criteria in this study ruled out most of the potential cofounding factors influencing the eGFR assessment. Fourth, the impacts of the different eGFR equations on clinical outcomes, complications, and other aspects of renal dysfunction (i.e., albuminuria and β_2_-microglobulin) and comparisons of the use of eGFR with the use of actual (reference) GFR were not explored. Further studies are warranted.

Taken together, we propose that the BSA-unadjusted CKD-EPI formula is the most favorable eGFR equation in patients with cancer, followed by the CKD-EPI and the Janowitz and Williams equations. Further validation studies with pharmacokinetic exploration are of interest.
